# Anesthetic management for resection of a massive tracheal tumor via combined electronic bronchoscopy and rigid endoscopy: A case report

**DOI:** 10.1097/MD.0000000000046410

**Published:** 2026-05-12

**Authors:** Yuanyuan Rong, Haijiao Wang, Zhaolei Tian, Huanshuang Pei, Shasha Zhang, Jianfeng Fu, Tao Hu, Xuan Zhou, Huaqin Liu

**Affiliations:** a Department of Anesthesiology, The Fourth Hospital of Hebei Medical University, Shijiazhuang, Hebei Province, China; b Department of Clinical Laboratory, Fourth Hospital of Shijiazhuang, Shijiazhuang, China.

**Keywords:** anesthesia, case report, tracheal tumor, ventilation

## Abstract

**Rationale:**

Tracheal tumors are a relatively uncommon pathology, with the majority being malignant, accounting for approximately 75% of all tracheal neoplasms. These tumors can lead to airway narrowing and obstruction, resulting in significant respiratory symptoms such as dyspnea. This case report discusses a patient with a tracheal tumor located at the carina, which caused complete obstruction of the left main bronchus and severe narrowing of the right main bronchus.

**Patient concerns:**

The patient presented with acute dyspnea due to the significant airway obstruction caused by the tumor.

**Diagnoses:**

Clinical evaluation, along with imaging studies, confirmed the presence of a tracheal tumor located at the carina, obstructing the left main bronchus and severely narrowing the right main bronchus.

**Interventions:**

The obstruction was managed through a staged approach. Initial interventions included partial tumor resection via flexible bronchoscopy under conscious sedation using dexmedetomidine and nalbuphine, followed by rigid bronchoscopy under general anesthesia using propofol and remimazolam for complete tumor debulking.

**Outcomes:**

Following the interventions, the patient experienced a resolution of dyspnea, and the airway was successfully restored, leading to a positive clinical outcome.

**Lessons:**

This case illustrates the importance of a staged anesthetic approach in complex airway management, particularly in the context of airway obstruction due to tracheal tumors. Effective anesthesia management facilitated successful tumor removal, highlighting the critical role of comprehensive patient assessment and tailored interventions in improving respiratory function and outcomes.

## 1. Introduction

Airway tumors can lead to severe airway stenosis and progressive obstruction, which may be life-threatening if effective ventilation cannot be established after the induction of general anesthesia.^[1]^ During trachelectomy, the shared airway necessitates the maintenance of ventilation while preserving surgical access within the trachea.^[2]^ Successful perioperative anesthesia management hinges on comprehensive preoperative evaluation, accurate problem anticipation, and formulation of a tailored airway management strategy.^[3]^

## 2. Case report

A 57-year-old male (height, 172 cm; weight, 70 kg) presented with a 2-week history of cough, sputum production, chest tightness, and dyspnea without apparent triggers. He had no previous diagnoses of hypertension, diabetes mellitus, or coronary heart disease, but had undergone right lower limb immobilization 20 years previously due to a foot injury. After admission, the patient’s vital signs were as follows: blood pressure, 124/97 mm Hg; heart rate, 94 beats/min; respiratory rate, 20 breaths/min; and temperature, 36.0°C. The patient was diagnosed with a tracheal mass, and tumor resection using a rigid bronchoscope was proposed.

Postadmission auxiliary examinations, including routine blood tests, biochemical analyses, and coagulation function tests, did not reveal any obvious abnormalities. Deep venous ultrasonography of both lower extremities and echocardiography did not reveal any significant pathological findings. Electrocardiography indicated sinus tachycardia.

**Figure 1.**
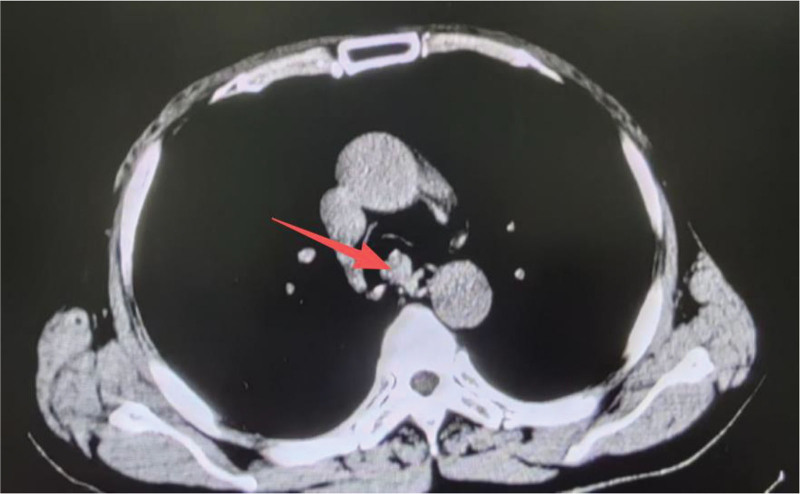
The tracheal tumor depicted on computed tomography, indicated by the red arrow.

**Figure 2.**
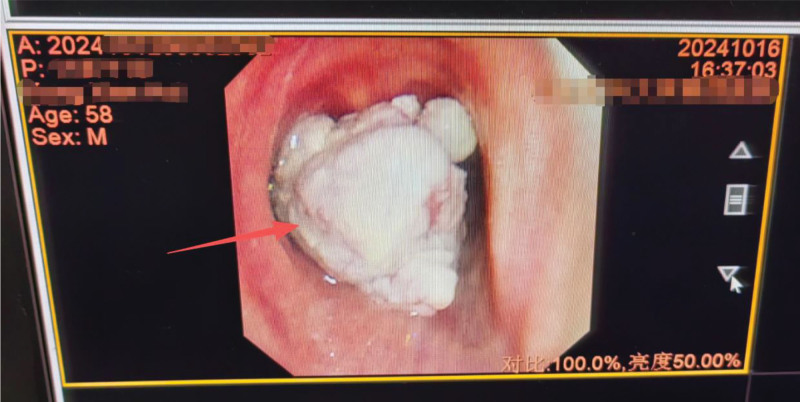
The tracheal tumor, as observed under bronchoscopy, indicated by the red arrow.

Computed tomography (CT) imaging (Fig. 1) revealed a smooth trachea and main bronchial openings, with a distinct soft tissue mass protruding into the tracheal lumen, measuring approximately 1.7 cm × 1.7 cm, and demonstrating contrast enhancement. Additionally, ground-glass opacities, approximately 5 mm × 5 mm in size, were observed in the anterior basal segment of the left lower lung lobe. Bronchoscopic examination (Fig. 2) confirmed a patent trachea with smooth mucosa, a well-defined bulge, and a cauliflower-like neoplasm obstructing the lumen. The right main bronchus had a narrow opening with smooth segmental orifices and no visible neoplasm. However, the left main bronchus was obstructed by a neoplastic lesion, which prevented complete bronchoscopic evaluation and resulted in decreased oxygen saturation (SpO_2_) during the procedure, necessitating termination of the examination.

**Figure 3.**
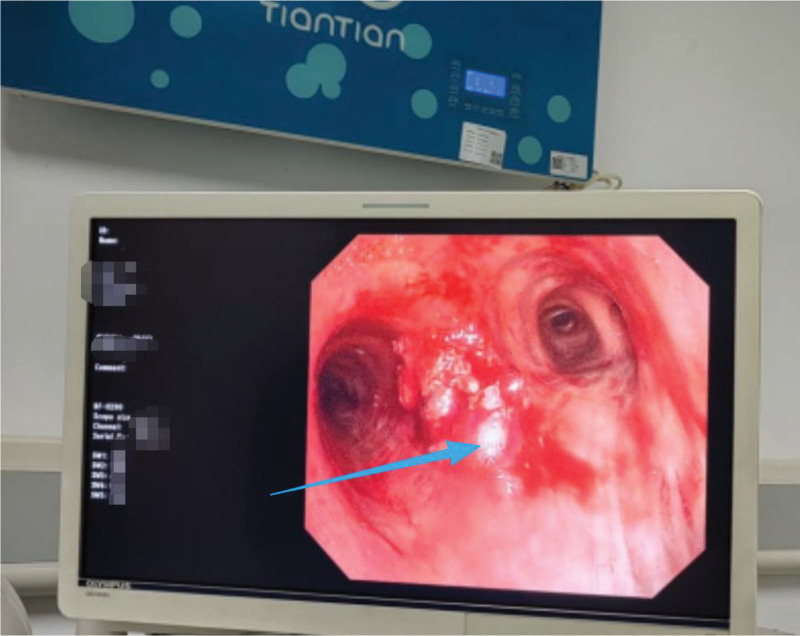
The tracheal carina after tracheal tumor resection, indicated by the green arrow.

**Figure 4.**
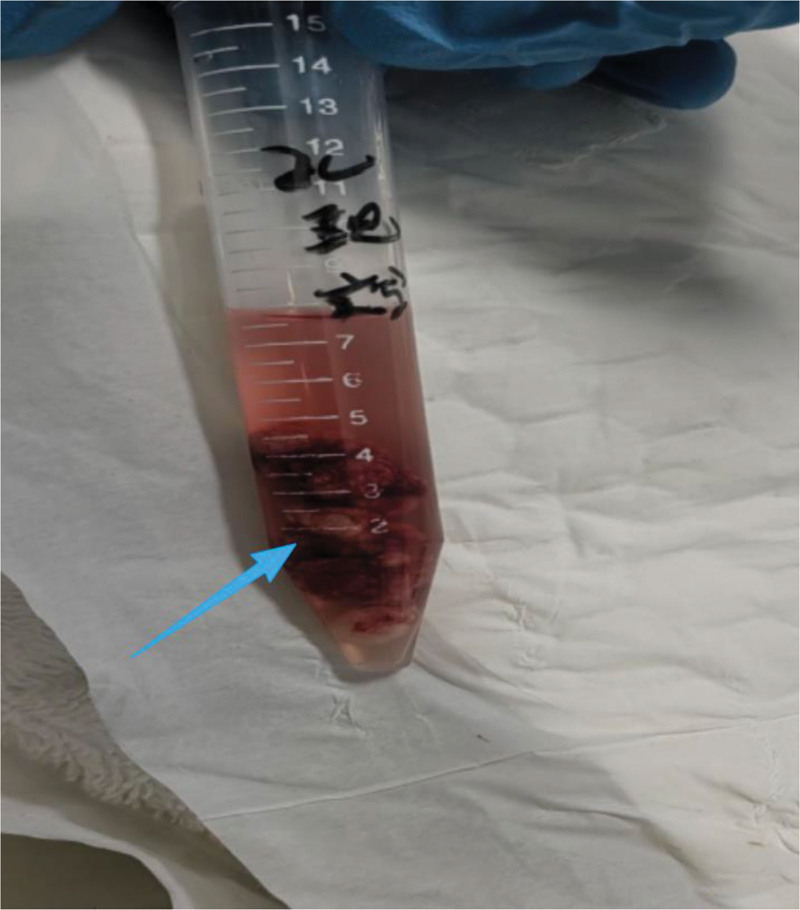
The resected tracheal tumor, indicated by the red arrow.

The patient presented with wheezing, orthopnea, and dyspnea. Vital signs included hypertension (blood pressure, 177/99 mm Hg), tachycardia (heart rate, 128 beats/min), and hypoxemia (SpO_2_ 94% on supplemental oxygen). Bronchoscopic evaluation revealed a tumor occluding the left main bronchus and severe narrowing of the right main bronchus. Given the airway findings, the surgeon determined the anesthetic management and surgical approach after electronic bronchoscopic exploration. The anesthetic strategy included topical anesthesia using 2 mL of 2% lidocaine administered via the nasal route. Subsequently, 10 mL oral dacronin gel paste was administered under oropharyngeal anesthesia. Subsequently, cricothyroid puncture was performed using 5 mL of 2% lidocaine for endotracheal topical anesthesia, followed by ultrasound-guided bilateral superior laryngeal nerve block with 1.5 mL of 2% lidocaine per side while maintaining the bispectral index between 70 and 80. Finally, intravenous anesthesia was induced using dexmedetomidine hydrochloride (10 μg) and nalbuphine hydrochloride (2 mg) while maintaining a bispectral index between 40 and 60. Approximately 3 minutes after the induction of anesthesia, the patient presented with severe stridor and a decrease in blood SpO_2_ to 94%, indicating imminent risk of airway compromise. Following thorough evaluation by an anesthesiologist and surgeon, a phased approach to mass resection was chosen, with initial electronic bronchoscopy performed under light anesthesia, followed by rigid bronchoscopic mass excision under deep anesthesia. An electronic bronchoscope was advanced from the tracheoscopic mask through the nasal cavity into the airway. After multiple irrigations of each bronchus, drops of 2% lidocaine were administered. Suction was then performed to remove the sputum and airway secretions, which revealed a rudimentary neoplasm that visibly obstructed the lumen of the left main bronchus and partially obstructed the right main bronchus. The neoplasms appeared brittle and bled easily upon contact. The mass was partially excised using electrocollaring, and the excised tissue was removed by freezing. Removal of the mass resulted in significant reduction in airway obstruction, which in turn led to an increase in blood SpO_2_ to 97%. With alleviation of the patient’s dyspnea, the surgeon opted to proceed with the second stage of resection to address the remaining mass under rigid tracheoscopy. Given the intense irritation caused by rigid tracheoscopy, the anesthesiologist administered 70 mg propofol to deepen the anesthesia. Additionally, 5 mL of 2% lidocaine was topically applied to the trachea for surface anesthesia. Due to pronounced respiratory depression associated with propofol, maintenance anesthesia was transitioned to intermittent dosing of remazolam (5 mg per administration), totaling 20 mg. The surgeon made numerous electrocautery incisions using a coiler during rigid tracheoscopy, removing most of the mass through cryotherapy, followed by electrocoagulation to control bleeding (during electrocoagulation, oxygen levels were maintained below 40% to prevent combustion). The surgery proceeded smoothly and lasted approximately 30 minutes, resulting in the removal of most of the mass (Figs. 3 and 4). A dose of flumazenil (0.5 mg) was administered at the conclusion of the procedure, leading to patient awakening within 3 minutes. The patient reported cessation of wheezing and the ability to lie flat. On day 2 of follow-up, no adverse symptoms were reported, and the patient was discharged on postoperative day 4.

## 3. Discussion

Primary tracheal neoplasms are uncommon, with malignant tumors comprising the majority of cases, typically squamous cell carcinomas or adenoid cystic carcinomas, which account for 75% of all tracheal tumors.^[4]^ The anesthetic management of tracheal resection is exceptionally challenging, necessitating the navigation of high-risk factors associated with airway stenosis and shared airway anatomy. The risks associated with surgery and anesthesia in these patients are substantial, and even minor mismanagement can precipitate severe airway obstruction and life-threatening complications. Therefore, a multidisciplinary approach with careful selection of the surgical strategy and anesthetic plan tailored to individual patients is essential.

Therapeutic bronchoscopy, including techniques, such as laser (Nd:YAG and CO_2_), photodynamic therapy, cryotherapy, endobronchial brachytherapy, and stenting, is used to alleviate nonresectable tumors.^[5]^ Regardless of the airway mass removal technique, ensuring effective patient ventilation remains paramount. Traditionally, this involves positioning the tracheal tube over the tumor or its tip while maintaining spontaneous breathing. Currently, alternative methods, such as high-frequency jet ventilation,^[6]^ rigid bronchoscopy with tumor resection,^[7]^ a combination of Fogarty catheter with Fogarty bronchoscope and endotracheal tube,^[8]^ laryngeal mask airway,^[9]^ and extracorporeal circulation, are available to enhance ventilation and oxygenation.

The patient presented with a remarkably large (1.7 cm × 1.7 cm), friable, and hemorrhage-prone intra-airway mass accompanied by severe tracheal stenosis and pronounced wheezing symptoms. Using the conventional tracheal intubation technique carries a substantial risk for life-threatening complications, such as bleeding and complete airway obstruction due to tumor dislodgement, during the procedure. Conversely, the use of a laryngeal mask for general anesthesia may compromise access to the operative field. These unique features necessitate careful consideration of the anesthetic and surgical management to ensure patient safety and successful interventions. Management of this complex case involved a multidisciplinary approach involving anesthesiologists, pulmonologists, and bronchoscopists. The tumor was removed to preserve spontaneous respiration. In the first stage, a portion of the tumor was resected using a flexible bronchoscope under light anesthesia to relieve airway obstruction because flexible bronchoscopes cause less irritation to the airways. In the second stage, the majority of the remaining tumor was removed using a rigid bronchoscope with the patient under deep anesthesia, which provided greater access but was more stimulating to the airway.^[10]^ Bronchoscopy was performed under light anesthesia with mild sedation and analgesia, combined with nasal-oral-endotracheal surface anesthesia and supraglottic nerve block. This initial procedure removed a portion of the tumor, alleviated the patient’s dyspnea, and enabled the subsequent use of rigid bronchoscopy. In the second stage, deeper anesthesia was administered to remove most of the tumor via rigid bronchoscopy, thereby facilitating further surgical treatment. The combined use of flexible and rigid bronchoscopies in this case enabled the complementary advantages of each technique, overcoming the limitations of either approach alone and ensuring optimal patient safety. This novel treatment strategy for endotracheal tumors provides patients with effective ventilation and oxygenation without compromising the airway, thereby creating the necessary space for surgical intervention. Tailored application of anesthesia at different stages of the procedure represents another viable option for managing patients with airway neoplasms.

Ultrasound-guided superior laryngeal nerve block has been demonstrated to substantially mitigate airway irritation, apnea, and hypoxemia during treatment.^[11]^ Accordingly, this technique was used in the present case to attenuate the risk for hypoxemia. Ultrasound-guided superior laryngeal nerve blocks can effectively optimize patient management by blocking laryngeal sensation, reducing airway irritation, inhibiting laryngeal reflexes, and decreasing the required doses of propofol and remimazolam.^[12]^

Addressing the critical complications of stridor and desaturation that occur during the initial light sedation phase is important. This event highlights the precarious and dynamic nature of near-total airway obstruction, in which even minimal sedation can perturb the delicate balance in airflow. It would be inaccurate to describe a course in which this occurs as entirely “stable.” Therefore, the success of anesthetic management in this case should be redefined –not by the absence of challenges – but by the multidisciplinary team’s preparedness for them. Our preoperative planning included specific protocols for such an eventuality, which enabled rapid recognition, discontinuation of the aggravating maneuver, and immediate transition to the staged approach. This adaptability is fundamental to a positive outcome and prevents life-threatening complete obstruction.

Multiple emergency treatment plans should be in place for potential complications during surgery. First, if the mass bleeds or detaches, causing airway obstruction, it can be maneuvered into one bronchus using a tracheoscope, enabling quick tracheal tube insertion under tracheoscopic guidance, ensuring patient ventilation before mass removal. Second, if airway obstruction from laxity postanesthesia impedes ventilation, a thinner wire-bearing catheter should be used to secure ventilation and patient safety. Third, in this case, rigid tracheoscopy enabled high-frequency or constant-frequency jet ventilation via the side port. Fourth, a tracheotomy set should be ready for immediate use, with otorhinolaryngologists available for collaborative diagnosis and treatment. Fifth, before the procedure, a multidisciplinary team discussion was held, and veno-venous extracorporeal membrane oxygenation was identified as the primary rescue strategy in the event of irreversible airway collapse or catastrophic hemorrhage. The cardiac surgery team and perfusionists were placed on immediate standby and the operating room was prepared for rapid cannulation.

The decision to avoid primary general anesthesia with endotracheal intubation was deliberate. While offering a secure airway and controlled ventilation, attempted tube passage past this large, friable carinal mass carries an unacceptable risk for iatrogenic catastrophe, including tumor dislodgement, hemorrhage, or conversion of a critical stenosis into a complete obstruction, resulting in a “cannot intubate, cannot ventilate” scenario. Furthermore, an indwelling tube can significantly obstruct surgical access. Alternative techniques, such as jet ventilation, were considered, but were deemed risky due to potential gas trapping distal to the obstruction. Our staged approach, which initially preserves spontaneous ventilation to visually assess and partially debulk the lesion under light sedation, prioritizes the avoidance of iatrogenic harm. This created a safer pathway to subsequently induce general anesthesia and proceed with definitive resection via rigid bronchoscopy, which served as a secure airway.

## 4. Conclusions

The case described herein underscores the significance of tailored airway management strategies for patients with sizeable endotracheal neoplasms. Using a staged approach, which combined light anesthesia with flexible bronchoscopy and deep anesthesia with rigid bronchoscopy, ensured patient safety and successful completion of the procedure. Furthermore, the use of ultrasound-guided supraglottic nerve block mitigated airway irritation and reduced the required anesthetic dosage.

The staged anesthetic management and supraglottic nerve block described herein offer valuable insights into the perioperative care of patients with airway masses. Emphasis was placed on intraoperative preparedness for emergency airway management. Sound preoperative planning, comprehensive contingency strategies, adaptable anesthetic protocols, and multidisciplinary collaboration are essential for the anesthetic management of these complex cases.

## Acknowledgments

The authors thank Huaqin Liu, from the Department of Anesthesiology of the Fourth Hospital of Hebei Medical University, who assisted in creating the figures.

## Author contributions

**Conceptualization**: Xuan Zhou.

**Formal analysis**: Huanshuang Pei.

**Investigation**: Zhaolei Tian.

**Methodology**: Haijiao Wang, Huaqin Liu.

**Supervision**: Jianfeng Fu.

**Validation**: Tao Hu.

**Writing – original draft**: Shasha Zhang.

**Writing – review & editing**: Yuanyuan Rong.
